# Multiple-center evaluation of mortality associated with acute kidney injury in critically ill patients: a competing risks analysis

**DOI:** 10.1186/cc10241

**Published:** 2011-05-17

**Authors:** Christophe Clec'h, Frédéric Gonzalez, Alexandre Lautrette, Molière Nguile-Makao, Maïté Garrouste-Orgeas, Samir Jamali, Dany Golgran-Toledano, Adrien Descorps-Declere, Frank Chemouni, Rebecca Hamidfar-Roy, Elie Azoulay, Jean-François Timsit

**Affiliations:** 1 Medical-Surgical Intensive Care Unit, Avicenne Teaching Hospital, 125 Route de Stalingrad, F-93009 Bobigny Cedex, France; 2 INSERM U823, Clinical Epidemiology of Critically Ill Patients and Airway Cancer, Albert Bonniot Institute, Rond-Point de la Chantourne, BP 217, F-38043 Grenoble, France; 3 Medical Intensive Care Unit, Gabriel Montpied Teaching Hospital, 58 Boulevard Montalembert, F-63003 Clermont-Ferrand Cedex 1, France; 4 Medical-Surgical Intensive Care Unit, Saint-Joseph Hospital, 185 Rue Raymond Losserand, F-75014 Paris, France; 5 Medical-Surgical Intensive Care Unit, Dourdan Hospital, 2 rue du Potelet, BP 102, F-91415 Dourdan Cedex, France; 6 Medical-Surgical Intensive Care Unit, Gonesse Hospital, 25 rue Pierre de Theilley, BP 30071, F-95503 Gonesse France; 7 Surgical Intensive Care Unit, Antoine Béclère Teaching Hospital, 157 rue de la Porte de Trivaux, F-92141 Clamart Cedex, France; 8 Medical Intensive Care Unit, Albert Michallon Teaching Hospital, BP 217, F-38043 Grenoble Cedex 09, France; 9 Medical Intensive Care Unit, Saint-Louis Teaching Hospital, 1 rue Claude Vellefaux, F-75010 Paris, France

## Abstract

**Introduction:**

In this study, we aimed to assess the association between acute kidney injury (AKI) and mortality in critically ill patients using an original competing risks approach.

**Methods:**

Unselected patients admitted between 1997 and 2009 to 13 French medical or surgical intensive care units were included in this observational cohort study. AKI was defined according to the RIFLE criteria. The following data were recorded: baseline characteristics, daily serum creatinine level, daily Sequential Organ Failure Assessment (SOFA) score, vital status at hospital discharge and length of hospital stay. Patients were classified according to the maximum RIFLE class reached during their ICU stay. The association of AKI with hospital mortality with "discharge alive" considered as a competing event was assessed according to the Fine and Gray model.

**Results:**

Of the 8,639 study patients, 32.9% had AKI, of whom 19.1% received renal replacement therapy. Patients with AKI had higher crude mortality rates and longer lengths of hospital stay than patients without AKI. In the Fine and Gray model, independent risk factors for hospital mortality were the RIFLE classes Risk (sub-hazard ratio (SHR) 1.58 and 95% confidence interval (95% CI) 1.32 to 1.88; *P *< 0.0001), Injury (SHR 3.99 and 95% CI 3.43 to 4.65; *P *< 0.0001) and Failure (SHR 4.12 and 95% CI 3.55 to 4.79; *P *< 0.0001); nonrenal SOFA score (SHR 1.19 per point and 95% CI 1.18 to 1.21; *P *< 0.0001); McCabe class 3 (SHR 2.71 and 95% CI 2.34 to 3.15; *P *< 0.0001); and respiratory failure (SHR 3.08 and 95% CI 1.36 to 7.01; *P *< 0.01).

**Conclusions:**

By using a competing risks approach, we confirm in this study that AKI affecting critically ill patients is associated with increased in-hospital mortality.

## Introduction

Acute renal failure (ARF) is as an abrupt decline in kidney function. Although simple to define conceptually, there has long been no consensus on an operational definition of ARF. As reported in a recent survey, more than 35 definitions have been used so far [1]. Depending on the definition used, ARF has been shown to affect from 1% to 25% of intensive care unit (ICU) patients and has led to mortality rates from 15% to 60% [2].

Because the lack of a uniform definition is a major impediment to epidemiological research in the field, the Acute Dialysis Quality Initiative Group (ADQIG) [3] recently proposed consensus definition criteria, namely, the RIFLE criteria based on three grades of increasing severity (Risk of renal dysfunction, Injury to the kidney, and Failure of kidney function) and two outcome classes (Loss of kidney function and End-stage kidney disease) (Table 1). Furthermore, they proposed that the old nomenclature ARF be replaced by the term acute kidney injury (AKI) to encompass the entire spectrum of the syndrome, from minor changes in renal function to need for renal replacement therapy (RRT).

**Table 1:** RIFLE classification^a^

RIFLE class	GFR criteria	UO criteria
Risk	Increase in serum creatinine ≥1.5 × baseline or decrease in GFR ≥25%	<0.5 ml/kg/hour for ≥6 hours
Injury	Increase in serum creatinine ≥2 × baseline or decrease in GFR ≥ 50%	<0.5 ml/kg/hour for ≥12 hours
Failure	Increase in serum creatinine ≥3 × baseline or decrease in GFR ≥75% or serum creatinine ≥350 μmol/L with an acute rise of at least 44 μmol/L	<0.3 ml/kg/hour for ≥24 hours or anuria ≥12 hours
Loss	Complete loss of kidney function >4 weeks	
End-stage kidney disease	Need for RRT >3 months	

^a^GFR, glomerular filtration rate; UO, urine output; RRT, renal replacement therapy.

The RIFLE classification is undoubtedly a major advance in that it allows easier comparisons across studies. Overall, it seems to correlate well with patients' outcomes [4-9]. In the ICU setting, only four multiple-center studies using the RIFLE criteria have been published so far [10-13]. All but one [12] found AKI to be associated with a poor outcome, with some residual heterogeneity regarding both incidence and mortality, however. In addition, estimates of AKI-associated mortality in these studies derived from traditional logistic regression or Cox models, while concerns about their reliability have been raised recently [14]. Briefly, logistic regression analysis ignores the timing of events and their chronological order, potentially leading to an overestimation of the association between a specific risk factor (for example, nosocomial pneumonia) and mortality [15]. This problem can be solved to some extent by applying the Cox model, which allows for the consideration of time-dependent covariates. Yet, this model does not deal with the competing risks issue. This issue arises when more than one endpoint is possible [16]. Typically, "dying in hospital" and "discharge alive" are two competing risks. If "dying in hospital" is the event of interest, the nonfatal competing event "discharge alive" hinders the event of interest from occurring as a first event.

Statistical models able to handle time-dependent covariates and allowing the simultaneous analysis of different endpoints (that is, competing risks) are now available [15,17-19]. In recent years, these models have engendered growing interest in hospital epidemiology (especially with regard to cancer research) but have rarely been used in the ICU field.

The aim of this study was to further assess the association between AKI defined by RIFLE criteria and in-hospital mortality in critically ill patients by using such an original competing risks approach.

## Materials and methods

### Study design and data source

We conducted an observational study in a multiple-center database (OUTCOMEREA) from January 1997 to June 2009. The methods of data collection and the quality of the database have been described in detail elsewhere [20]. Briefly, the database receives information from 13 French ICUs. To avoid selection bias and ensure external validity, a random sample of patients older than 16 years of age and staying in the ICU for >24 hours are entered into the database each year. Participating centers can choose between two modes of patient selection: (1) consecutive admissions in "n" ICU beds for the whole year or (2) consecutive admissions in a particular month. The allocation of beds (or a particular month) is decided yearly by the database's steering committee.

Data are prospectively collected on a daily basis by senior physicians of the participating ICUs who are closely involved in establishing the database. For all patients, information is recorded at baseline (including demographic characteristics, comorbidities, baseline severity, admission diagnosis, admission category and transfer from ward) and on each consecutive day throughout the ICU stay (including diagnostic and therapeutic procedures, biological parameters, organ failure, sepsis, occurrence of iatrogenic events and decision to withhold or withdraw life-sustaining treatments). The quality control procedure involves multiple automatic checking of internal consistency and biennial audits. Moreover, a one-day data capture training course is held once yearly for all OUTCOMEREA investigators and study monitors. OUTCOMEREA senior physicians and participating centers are listed in the Acknowledgements.

In accordance with French law, the development and maintenance of the OUTCOMEREA database were disclosed to the Commission Nationale de l'Informatique et des Libertés. The study was approved by the ethics committee of Clermont-Ferrand, France. Because routine collection of data entered into the database did not modify patients' management in any way, and because statistical analyses were processed anonymously, informed consent for participation in the study was waived.

### Study population and definitions

All patients in the database were eligible for inclusion in the study. For patients who were admitted more than once to the ICU, only the first ICU stay was included in the analysis. AKI was defined according to the RIFLE criteria. Patients were classified according to the maximum RIFLE class (no AKI, Risk, Injury or Failure) reached during their ICU stay as described in previous reports [10,11,13]. For patients who received RRT, the maximum RIFLE class was that reached before RRT initiation. Since the 6- and 12-hour urine outputs were not recorded in the database, we used the glomerular filtration rate (GFR) only. The GFR criteria were determined according to changes in serum creatinine level from baseline values. Because AKI may be present on ICU admission in a high proportion of patients, we chose to assess baseline creatinine values using the Modification of Diet in Renal Disease (MDRD) equation. As recommended by the ADQIG, a normal GFR of 75 ml/minute/1.73 m^2 ^before ICU admission was assumed [3].

Patients with chronic kidney disease (assessed according to the Acute Physiology and Chronic Health Evaluation (APACHE) II definitions [21]) and patients with a nonorganic (prerenal) cause of renal dysfunction (identified by a specific code in the database) were excluded because their prognosis is potentially different (better) from that of patients with "true" *de novo *organic AKI. Patients put on RRT while no diagnosis of AKI had been made (that is, patients with RRT for "extrarenal indications" such as intoxications or cardiogenic shock) were also excluded because it was impossible to determine whether AKI was not actually present or could not be diagnosed thereafter as a consequence of the reduction in serum creatinine due to RRT. Finally, any decision to withhold or withdraw life-sustaining treatments led to exclusion of patients from analysis to avoid biasing the estimation of the association between AKI and hospital mortality.

### Data collection

The following data were recorded:

**1. **Upon ICU admission: patient age, sex, McCabe class (class 1, no fatal underlying disease; class 2, underlying disease fatal within 5 years; class 3, underlying disease fatal within 1 year [22]) Simplified Acute Physiology Score (SAPS) II, nonrenal Sequential Organ Failure Assessment (SOFA) score (SOFA renal component), comorbidities assessed according to the Acute Physiology and Chronic Health Evaluation (APACHE) II definitions, transfer from ward (defined as a stay in an acute bed ward ≥24 hours immediately before ICU admission) and admission category (medical, scheduled surgery, or unscheduled surgery).

2. During the ICU stay: daily serum creatinine level, time from admission to occurrence of AKI, time from admission to the maximum RIFLE class and daily SOFA score.

3. Upon ICU discharge: length of ICU stay.

4. Upon hospital discharge: length of hospital stay and vital status.

### Endpoints

The primary endpoint was hospital mortality. The secondary endpoints were the length of ICU stay and hospital stay.

### Statistical analyses

Comparisons of patients with and those without AKI were based on χ^2 ^tests for categorical data and on Student's *t*-test or Wilcoxon's rank-sum test for continuous data as appropriate. Comparisons of AKI patients according to their maximum RIFLE class were based on χ^2 ^tests for categorical data and on one-way analysis of variance or the Kruskal-Wallis test for continuous data as appropriate.

The association of AKI with mortality was assessed according to the Fine and Gray [23] subdistribution hazard regression model, which extends the Cox model to competing risk data by considering the hazard function associated with the cumulative incidence function (CIF). The main advantage of the CIF and Fine and Gray model over the Kaplan-Meier (KM) method and Cox model pertains to censoring. Indeed, the KM method and the Cox model assume that censoring is uninformative (that is, that the survival time of an individual is independent of censoring). Accordingly, patients discharged alive at time *t *are considered to be representative of all other patients who have survived to this time *t *but who still have not been discharged. This may be true when the censoring process operates randomly. However, this assumption probably cannot be made in the case of ICU patients. Actually, since these patients are discharged alive (censored) because of an improvement (or sometimes a deterioration) of their medical state, they have a lower (or sometimes higher) risk of dying than the average and are therefore not representative of other patients who have not been censored yet. Thus, censoring is clearly informative (that is, the survival time of an individual does depend on censoring). In other words, informative censoring defines a competing risk, given that discharge alive affects the probability of experiencing the event of interest (death before discharge). In this setting, standard survival methods are no longer valid, and specific approaches, such as the CIF and Fine and Gray model that allow handling of both time to events and informative censoring [24,25], merit consideration.

At time *t*, the CIF defines the probability of dying, provided that the study population has survived at time *t *-1. Contrary to a distribution function that tends toward 1, the CIF tends to the raw proportion of deaths. Thus it is also called "subdistribution function". The strength of the association between a specific risk factor and the event of interest in the Fine and Gray model is reflected by the sub-hazard ratio (SHR), which is the ratio of hazards associated with the CIF in the presence and absence of the risk factor. Note that this model was originally developed for time-independent risk factors [23]. However, while cumulative incidence is no longer available for time-dependent risk factors, cumulative hazards may be considered instead and SHR can still be computed [26].

We first computed SHR for mortality and 95% confidence intervals associated with each of the Risk, Injury and Failure classes in univariate analysis. Then we performed a multivariate analysis to adjust for the following predefined potential confounding factors: baseline characteristics (nonrenal SOFA score, McCabe class, admission category and transfer from ward) and other organ failures (assessed on the basis of a specific SOFA component >2) occurring before AKI. To account for their timing and chronological order [26], each RIFLE class and organ failure were entered into the Fine and Gray model as time-dependent variables (in other words, time to organ failure and changes over time were implicitly considered).

A *P *value < 0.05 was considered significant. Analyses were computed using the SAS 9.1 software (SAS Institute, Cary, NC, USA) and the free R software package.

## Results

### Study population

**Figure 1:**
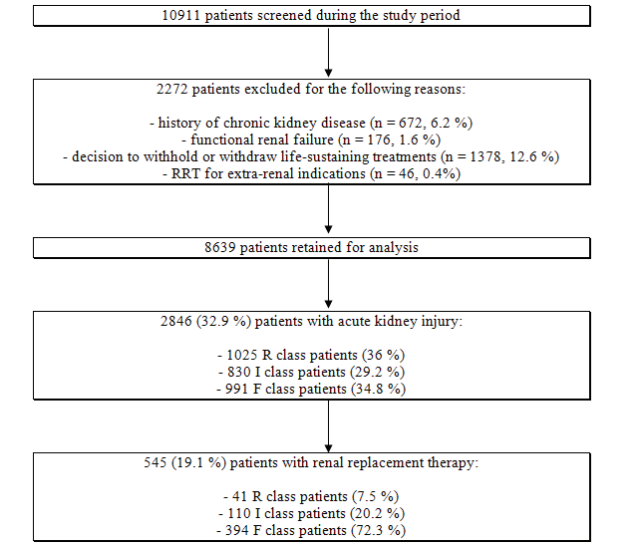
**Study flow chart**. RRT, renal replacement therapy; R class, Risk; I class, Injury; F class, Failure.

Of the 10,911 patients in the OUTCOMEREA database, 2,272 (20.8%) had exclusion criteria. Among the remaining 8,639 patients, 2,846 (32.9%) had AKI, of whom 545 (19%) received RRT (Figure 1).

Patients with AKI were older, had higher severity scores, were more likely to have undergone unscheduled surgery and had more severe comorbidities than patients without AKI (Table 2). Among AKI patients, higher severity scores and unscheduled surgery were associated with a higher degree of renal dysfunction (Table 3).

**Table 2:** Baseline characteristics of patients with and those without AKI^a^

Variable	Patients with AKI (*n *= 2,846)	Patients without AKI (*n *= 5,793)	*P *value
Mean age, years (±SD)	66.4 (15.9)	55.6 (18.5)	<0.0001
Males, *n *(%)	1,672 (58.8)	3,609 (62.3)	0.002
Mean SAPS II score (±SD)	50.2 (20.0)	33.6 (16.9)	<0.0001
Mean APACHE II score (±SD)	19.9 (7.1)	12.9 (6.4)	<0.0001
Mean non-renal SOFA score (±SD)	5.3 (3.2)	3.6 (2.7)	<0.0001
Transfer from ward, *n *(%)	1363 (47.9)	2494 (43.1)	<0.0001
McCabe class, *n *(%)			
1	1,666 (58.5)	4,074 (70.3)	<0.0001
2	959 (33.7)	1,417 (24.5)	
3	221 (7.8)	302 (5.2)	
Admission category, *n *(%)			
Medical	2,043 (71.8)	4,149 (71.6)	<0.0001
Scheduled surgery	311 (10.9)	865 (14.9)	
Unscheduled surgery	492 (17.3)	779 (13.5)	
Chronic coexisting conditions, *n *(%)			
Cardiac disease	509 (17.9)	497(8.6)	<0.0001
Respiratory disease	366 (12.9)	881 (15.2)	0.004
Liver disease	178 (6.3)	288 (5.0)	0.01
Immunodeficiency	440 (15.5)	688 (11.9)	<0.0001
Uncomplicated diabetes mellitus	320 (11.2)	431 (7.4)	<0.0001
Complicated diabetes mellitus	148 (5.2)	124 (2.1)	<0.0001

^a^AKI, acute kidney injury; SAPS, Simplified Acute Physiology Score; APACHE, Acute Physiology and Chronic Health Evaluation; SOFA, Sequential Organ Failure Assessment; nonrenal SOFA: SOFA renal component.

**Table 3:** Baseline characteristics of AKI patients according to the maximum RIFLE class reached during the intensive care unit stay^a^

Variable	Class R patients (*n *= 1,025)	Class I patients (*n *= 830)	Class F patients (*n *= 991)	*P *value
Mean age, years (±SD)	67.6 (15.8)	66.7 (15.7)	64.9 (16.0)	<0.001
Males, *n *(%)	588 (57.4)	502 (60.5)	582 (58.7)	0.4
Mean SAPS II score (±SD)	45.2 (17.4)	51.9 (21.2)	53.8 (20.3)	<0.0001
Mean APACHE II score (±SD)	18 (6.6)	20.6 (7.1)	21.4 (7.1)	<0.0001
Mean non-renal SOFA score (±SD)	4.8 (3.1)	5.8 (3.3)	5.4 (3.4)	<0.0001
Transfer from ward, *n *(%)	477 (46.5)	387 (46.6)	499 (50.4)	0.16
McCabe class, *n *(%)				
1	608 (59.3)	476 (57.3)	582 (58.7)	0.8
2	342 (33.4)	290 (35.0)	327 (33)	
3	75 (7.3)	64 (7.7)	82 (8.3)	
Admission category, *n *(%)				
Medical	754 (73.6)	592 (71.3)	697 (70.3)	<0.002
Scheduled surgery	130 (12.7)	85 (10.2)	96 (9.7)	
Unscheduled surgery	141 (13.8)	153 (18.4)	198 (20.0)	
Chronic coexisting conditions, *n *(%)				
Cardiac disease	185 (18.1)	163 (19.6)	161 (16.3)	0.2
Respiratory disease	165 (16.1)	101 (12.2)	100 (10.1)	<0.001
Liver disease	61 (6.0)	59 (7.1)	58 (5.9)	0.5
Immunodeficiency	143 (14.0)	137 (16.5)	160 (16.2)	0.2
Uncomplicated diabetes mellitus	125 (12.2)	90 (10.8)	105 (10.6)	0.5
Complicated diabetes mellitus	45 (4.4)	40 (4.8)	63 (6.4)	0.1

^a^AKI, acute kidney injury; SAPS, Simplified Acute Physiology Score; APACHE, Acute Physiology and Chronic Health Evaluation; SOFA, Sequential Organ Failure Assessment; non-renal SOFA: SOFA renal component.

### Dynamics of AKI

AKI was a rapidly evolving process. Times from ICU admission to occurrence of AKI (median days (interquartile range)) were 1 (1 to 2), 2 (1 to 2) and 1 (1 to 2) in the class R, I and F patients, respectively. Times from ICU admission to maximum RIFLE class were 1 (1 to 2), 2 (1 to 3) and 2 (1 to 3) in R, I, and F patients, respectively.

**Figure 2:**
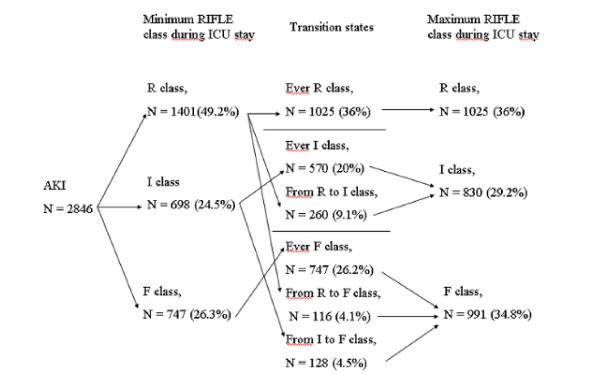
**Dynamics of acute kidney injury (AKI) during intensive care unit (ICU) stay**. The flowchart illustrates the lowest and highest degrees of renal dysfunction reached during the ICU stay and the proportion of patients displaying progressive alteration of kidney function.

Figure 2 illustrates the lowest and highest degrees of renal dysfunction reached during the ICU stay and the proportion of patients displaying progressive alteration of kidney function.

### Impact of AKI on mortality

Overall, hospital mortality rates were higher in patients with AKI than in those without AKI (27.6% vs. 8.7%; *P *< 0.0001). Among AKI patients, I and F class patients had higher mortality rates than R class patients (33.9% and 33.5% vs. 16.7%, respectively; *P *< 0.0001).

The multivariate Fine and Gray model revealed that R, I and F classes of the RIFLE criteria were independent risk factors for in-hospital mortality (Table 4). Other variables independently associated with in-hospital mortality were nonrenal SOFA score, McCabe class 3 and respiratory failure occurring before AKI onset (Table 4).

**Table 4:** Association of AKI with hospital mortality: results of the unadjusted and adjusted Fine and Gray models^a^

Variable	SHR univariate analysis (95% CI)	*P *value	SHR multivariate analysis (95% CI)	*P *value
No AKI	1	-	1	-
R class	2.28 (1.62 to 3.19)	<0.0001	1.58 (1.32 to 1.88)	<0.0001
I class	7.39 (5.37 to 10.17)	<0.0001	3.99 (3.43 to 4.65)	<0.0001
F class	9.73 (8.16 to 11.60)	<0.0001	4.12 (3.55 to 4.79)	<0.0001
Non-renal SOFA score, per point	-	-	1.19 (1.1.18 to 1.21)	<0.0001
McCabe class 3	-	-	2.71 (2.34 to 3.15)	<0.0001
Respiratory failure	-	-	3.08 (1.36 to 7.01)	<0.01

^a^SHR, sub-hazard ratio; 95% CI, 95% confidence interval; SOFA, Sequential Organ Failure Assessment; non-renal SOFA: SOFA renal component.

### Impact of AKI on lengths of stays and need for prolonged renal support

Patients with AKI had longer (median days (interquartile range)) ICU stays (no AKI: 4 (3 to 7), R class: 6 (3 to 11), I class: 7 (4 to 12) and F class: 8 (4 to 17), *P *< 0.001) and longer hospital stays (no AKI: 16 (9 to 30), R class: 22 (12 to 40), I class: 21 (10 to 37) and F class: 25 (12 to 44); *P *< 0.001) than patients without AKI. Upon ICU discharge, 92 survivors (3.2%) among the 2,846 AKI patients still needed renal support.

## Discussion

The association of AKI with critically ill patients' outcomes has been widely investigated, but very few multiple-center evaluations using the RIFLE criteria have been published so far [10-13]. Our study, carried out in a large cohort of general ICU patients, supports the use of RIFLE as a classification tool and confirms previous evidence that AKI negatively influences patients' outcomes.

The originality of our results lies mainly in the original competing risks approach. This approach has many potential advantages over the commonly used logistic regression and Cox models. Actually, logistic regression has been reported to cause loss of information because it yields a time-independent probability of dying and ignores the timing of events and their chronological order [27,28]. While the Cox model may partially alleviate these limits, it has been shown to overestimate the incidence of the event of interest, with most of the overestimation being related to the rate of the competing event [29]. By contrast, the Fine and Gray model adequately addresses time spent in the hospital as a risk factor for mortality by considering death hazard rates and takes into account the time-varying exposure status, thus avoiding a potential misjudgment in terms of time-dependent bias [30,31]. Moreover, it provides a more accurate estimation of mortality because death hazard rates are not confounded by the competing event "discharge alive."

In keeping with the few similar multiple-center evaluations that have used the RIFLE criteria [10,11,13], we found that AKI was an overall predictor of poor outcomes (it must be noted, however, that results regarding crude hospital mortality rates vary considerably from one study to another, indicating residual heterogeneity despite the use of consensual definition criteria) and that mortality differed according to the maximum RIFLE class reached during the ICU stay. Of note, even moderate renal dysfunction (R class) impaired patients' prognosis as previously shown [10,13,32], and, interestingly, the SHRs for I and F classes were similar. These data suggest, similarly to the study by Ostermann *et al*. [13], that the maximum risk of death might be reached as soon as patients are in I class of the RIFLE criteria. Thus, therapeutic and preventive strategies, such as optimization of hemodynamic parameters and avoidance of nephrotoxic drugs, must undoubtedly be in order at an early stage of renal dysfunction to prevent further aggravation and to reduce the risk of death.

Despite its strengths, our study has potential limitations. First, the definition of AKI was not based on the most recent consensus criteria proposed by the Acute Kidney Injury Network (AKIN) group [33]. The main differences between the AKIN and RIFLE classifications are as follows: a smaller change in serum creatinine level (>26.2 μmol/L) used to identify patients with stage 1 AKI (analogous to the RIFLE Risk class), a time constraint of 48 hours for the diagnosis of AKI and any patient receiving RRT classified as having stage 3 AKI. However, compared to the RIFLE criteria, there is currently no evidence that the AKIN criteria improve the sensitivity, robustness and predictive ability of the definition and classification of AKI in the ICU [34-36]. This is consistent with our finding that maximum renal dysfunction during the ICU stay was reached within a two-day period in most patients. Furthermore, classifying any patient receiving RRT in stage 3 is questionable and may introduce bias because of the lack of uniform recommendations regarding the timing and modalities of RRT.

Second, assessing baseline creatinine values by the MDRD equation as in previous reports may have exposed our study methodology to the risk of inclusion of patients with modest chronic disease not captured by the APACHE II definitions as having end-stage renal disease or RRT dependence. This is a potential source of misclassification bias [37] and underestimation of the association between AKI and hospital mortality. This issue needs further investigation.

Third, we encountered the same problem as others did [9,13]: the 6- and 12-hour urine outputs were not recorded in our database. Therefore, patients were classified according to the GFR criteria only. Patients classified according the GFR criteria seem to be more severely ill and have slightly higher mortality rates than their counterparts classified according to the urine output criteria [11,38,39]. Consideration of both criteria may have resulted in a lowest estimation of the risk of death (and, conversely, a higher incidence of AKI).

Fourth, the potential confusing role of RRT was not evaluated. However, the extent to which RRT interferes with AKI patients' prognosis remains unclear, and practices regarding this technique vary widely from one institution to another. Consequently, considering RRT as a confounder could have led to hazardous conclusions. This issue deserves further specific evaluation.

Fifth, although it is multicentered, our database is not multinational. So, our population may not be representative of ICU patients in other countries. Nevertheless, the baseline characteristics, AKI incidence and proportion of patients receiving RRT were similar to those reported in previous studies [11,13].

Finally, we did not have any information as to the exact etiology of AKI, although sepsis was probably the commonest one. Of note, a recent study revealed that RIFLE classification can be used to evaluate the overall prognosis of septic patients, suggesting a close link between AKI and sepsis [40]. However, AKI often results from a combination of several risk factors whose respective contributions are difficult to determine. Whether any of these risk factors plays a preponderant role (or whether patients' prognosis differs according to the cause of AKI) remains unknown.

## Conclusions

While the prognosis for patients with AKI has long remained unclear because of the lack of a uniform definition, the recently published RIFLE criteria have facilitated epidemiological research in the field. Three multiple-center studies using conventional statistical models found an association between RIFLE class and mortality [10,11,13]. Original competing risks models reflecting "real life" more accurately are now available but are rarely used in the ICU setting. By applying such a model, this study confirms that AKI affecting critically ill patients is associated with increased mortality. However, further investigations focusing on the potential confusing role of RRT are warranted to better characterize the prognosis of AKI patients.

## Key messages

• The association of AKI with critically ill patients' outcomes has been widely investigated, but very few multiple-center evaluations using recent consensus definition criteria have been published so far. Our study, carried out on a large cohort of general ICU patients, supports the use of RIFLE as a classification tool and adds to the current limited evidence that AKI negatively influences patients' outcomes.

• By applying an original competing risks approach and considering AKI as a time-dependent variable, we likely provided a refined estimation of the association between AKI and mortality as compared to previous reports.

• Further investigations focusing on the potential confusing role of RRT are warranted to better characterize the prognosis of AKI.

## Abbreviations

AKI: acute kidney injury; APACHE: Acute Physiology and Chronic Health Evaluation; ARF: acute renal failure; CIF: cumulative incidence function; GFR: glomerular filtration rate; ICU: intensive care unit; RIFLE: class R: Risk of renal dysfunction, class I: Injury to the kidney, class F: Failure of kidney function; class L: Loss of kidney function; and class E: End-stage kidney disease; RRT: renal replacement therapy; SAPS: Simplified Acute Physiology Score; SHR: subhazard ratio; SOFA: Sequential Organ Failure Assessment.

## Competing interests

The authors declare that they have no competing interests.

## Authors' contributions

CC designed the study and wrote the manuscript. CC, JFT and MNM performed the statistical analyses. FG, AL, MGO, SJ, DGT, ADD, FC, RHR and EA participated in the collection of data and critically revised the manuscript for important intellectual content. All authors read and approved the final manuscript.
